# Differences in Menopausal Hormone Therapy Use among Women in Germany between 1998 and 2003

**DOI:** 10.1186/1472-6874-7-19

**Published:** 2007-10-18

**Authors:** Yong Du, Martina Dören, Hans-Ulrich W Melchert, Christa Scheidt-Nave, Hildtraud Knopf

**Affiliations:** 1 Department of Epidemiology and Health Monitoring, Division of Non-Communicable Disease Epidemiology, Robert Koch-Institute, Berlin, Germany; 2 Clinical Research Centre for Women's Health, Charité-Universitätsmedizin Berlin, Campus Benjamin Franklin, Berlin, Germany

## Abstract

**Background:**

To examine the differences in menopausal hormone therapy (MHT) use and user profiles among women in Germany before and after the communication of the Women's Health Initiative (WHI) trial and other study results concerning the risks and benefits of MHT.

**Methods:**

Current MHT use was ascertained in two periodic German national health surveys conducted in 1997–1999 and 2003–2004. MHT prevalence and user profiles were assessed within each survey. The association of the survey period (2003–2004 vs. 1997–1999) with current MHT use was analyzed in weighted multivariable logistic regression (MLR) models, pooling data from both surveys.

**Results:**

The overall prevalence of current MHT use decreased by 40.2% from 16.9% of the sample in 1997–1999 to 10.1% in 2003–2004. The difference in prevalence between surveys varied with age decade with the smallest decreases among women 60–69 years of age (20.3% vs. 18.5%), compared to women of younger and older age groups (40–49: 10.7% vs. 3.9%; 50–59: 36.3% vs. 21.3%; 70–79: 5.7% vs. 3.2%). Variables independently associated with higher current MHT use in both health surveys included age category (curvilinear relationship with highest use among women 50–59 years) and residence in West vs. East Germany. A higher social status, lower body mass index, and more health-conscious behaviour were significantly associated with higher current MHT use in the 1997–1999 survey, but these associations were not found in the later survey. MLR analyses confirmed a significant decline in MHT use between the 1997–1999 and 2003–2004 surveys, however, the effect was modified by social status and was not significant among lowest social-status women.

**Conclusion:**

Current MHT use considerably declined among women in Germany between the pre- and post-WHI era. A convergence of current MHT use among women of higher social status with pre-existing patterns of use among lower social-status women suggests that MHT in Germany is now less likely to be used for health promotion.

## Background

During the past two decades menopausal hormone therapy (MHT, the term used for both estrogen therapy and estrogen plus progestin therapy) has been widely proposed and prescribed for postmenopausal women, not only for the relief of menopausal symptoms, but also for the prevention of cardiovascular diseases and osteoporosis in later life. Such advice to women was based on a wealth of supporting results from preclinical as well as observational clinical and epidemiological studies, although confirmatory data from randomized clinical trials (RCTs) were still lacking or not based on hard clinical endpoints [1,2]. Some experts kept advising caution in view of limited evidence [3]. However, it was not before the publication of results from two RCTs, the Heart and Estrogen/Progestin Replacement Study (HERS) in 1998 [4] and the combined estrogen plus progestin therapy arm of the Women's Health Initiative randomized controlled trial (EPT-WHI) in 2002 [5] as well as the large observational Million Women Study (MWS) in 2003 [6] that this position gained widespread attention among medical experts and health care officials [7,8]. Ever since, studies from the United States [9-16] and various other European countries [17-24], including Germany [25,26], have consistently reported a substantial decline in MHT use.

Up to now, few studies have examined changes in user profiles with respect to socio-demographic and co-morbidity patterns at a nationally representative level [15,19,23]. In the present study, we have analyzed differences in the prevalence and correlates of current MHT use among women in Germany, based on data from two periodic national health surveys conducted in 1997–1999 and 2003–2004.

## Methods

### Study design and population

The German National Health Interview and Examination Survey 1998 and the German National Health Telephone Survey 2004 were conducted by the Robert Koch-Institute between October 1997 and March 1999 (1997–1999 survey) and between September 2003 and March 2004 (2003–2004 survey). The designs, sampling frames and study protocols of both surveys have been previously described in detail [27,28]. In brief, two stage sampling procedures were applied in both surveys. In the 1997–1999 survey, a sample of German communities (80 in West Germany and 40 in East Germany) representing community size and structure in Germany was drawn at the first stage. Age- (5-year-intervals) and sex-stratified random samples of adult residents 18–79 years of age were then drawn from local population registries in proportion to the age and gender structure of the German adult population. The final sample included 7124 residents (3450 men; 3674 women) at a response rate of 61.4%. In the 2003–2004 survey, a pool of about 45,000 telephone numbers from complete listings of conventional telephone extensions belonging to private households in Germany was randomly generated, applying the Häder-Gabler method [29]. This method assures that households with unregistered telephone extensions are included in the 'target sample' of telephone surveys. Random sampling at the individual level was achieved by the 'next-birthday-method', i.e. only the adult whose birthday is coming up next to the date of first contact to the respective household is included in the target sample. Altogether, 56.1% of contacted persons 18 years of age and older completed the survey (3376 men; 3965 women). Menopause is most likely to occur between 40 and 60 years [30], and women in Germany tend to start MHT at a relatively young age [31]. Given a common upper age range of 79 years in the two surveys, the present analysis is therefore based on women who were 40–79 years at the time of participation. Thus, the study population includes a total of 2248 women participating in the 1997–1999 survey and 2215 women participating in the 2003–2004 survey.

Both surveys were approved by the Federal Office for the Protection of Data. Participants were informed in detail about the study objectives, interview and examination procedures, as well as pseudonymized record keeping and data analyses. Persons participating in the 1997–1999 survey gave written informed consent prior to the interview and examination, oral informed consent was sought in survey 2003–2004.

### Data assessment

In the 1997–1999 survey, information on health behaviour and socio-demographic background was collected by means of a self-administered questionnaire. Measures of height and weight, medical history and information on the use of medication were obtained as part of a highly standardized computer-assisted health interview and examination, conducted by specifically trained study physicians and assisting technical staff [27]. Use of any medication within the last seven days before the medical interview, including prescription drugs and over-the-counter medications, was assessed using a standardized drug use questionnaire. Details on the use of the medication, such as daily dose, route of application, and duration of use were documented. Participants were asked to bring original containers or package inserts to the examination site for the purpose of verification. Specific ATC-(Anatomical Therapeutic Chemical) codes were assigned to all reported medications. Based on this information, we were able to identify current MHT users as women currently taking sex hormone preparations used exclusively for MHT (ATC-codes G03C, G03D, G03E, G03F, G03HB).

Data collection in the 2003–2004 survey was based on a highly standardized computer-assisted telephone interview (CATI) [28]. The definition of MHT use was based on a sequence of two questions: 1) *Have you ever taken hormone preparations in connection with the menopause*? and 2) *Are you currently taking hormone preparations*? Women answering in the affirmative to both questions were defined as 'current MHT users'; if only question 1 was affirmed, women were defined as past MHT users.

Duration of MHT was assessed based on an ordinal scale ('≤ 1 year'; '1–3 years'; '> 3 years') in the 1997–1999 survey, as opposed to asking the number of years in the 2003–2004 survey. We therefore used '> 3 years' as a common definition of long-term MHT among current users. Neither survey questionnaire included a specific question to measure recent uptake of MHT. Using the available information, we estimated the prevalence of 'new starters' based on current MHT users who reported to have used MHT for up to one year.

Body mass index (BMI) in the 2003–2004 survey was computed from self-reported measures of weight and height, with adjustment for potential misclassification bias [32]. Apart from MHT use and BMI, only variables based on identical questions in both surveys were included in the analysis. A composite social-status index was computed, integrating educational level, household income and profession as previously described [33]. Scores from 1–7 were attributed to each of the components and the sum of the scores was calculated ranging from a possible minimum of 3 to a maximum of 21. Categories of social status were defined lower (3–8), intermediate (9–14), and upper (15–21) [33]. While based on identical questions to collect information on the individual components, the construct variable was adjusted to societal changes, namely income inflation and improvements in people's education. This assures a high degree of standardization with respect to data collection, as well as comparability of the construct over time (Robert Koch-Institute, unpublished data). A definition of pre-existing medical conditions, such as diabetes mellitus, hypertension, and hyperlipidemia was based on the question: *'Has a doctor ever told you that you have one of the following conditions?'*. Pre-existing cardiovascular disease (CVD) included a history of coronary heart disease, angina, congestive heart failure, previous myocardial infarction or stroke.

### Statistical analysis

A weighting factor was computed from survey-specific weighting factors adjusting for deviations in demographic characteristics (age, sex, residence in West or East Germany and level of urbanity) between the study populations and official population statistics at the time of the specific surveys [28,34] as well as at a point in time (2003) that served as a common standard for both surveys.

We used descriptive statistics to assess the main characteristics of the study population within each survey. The weighted prevalence of MHT and the distribution of socio-demographic and health-related co-variables were compared between the two surveys, applying chi-square and t-tests as appropriate. Survey specific multivariable logistic regression (MLR) analyses with current MHT status as the dependent variable and all other study characteristics as independent variables were performed to identify MHT user profiles within the two surveys. In order to estimate the independent effect of the survey period on current MHT use, a full model was fitted, pooling data from both surveys and adding the survey period (2003–2004 survey vs. 1997–1999 survey) as the independent variable of main interest. Socio-demographic characteristics, lifestyle variables and pre-existing morbidity as well as their first order interactions with the survey period were included as co-variables. As the interaction between survey period and social status was highly significant, the effect of the survey period on current MHT use was estimated in separate multivariate logistic regression models stratified by social status.

All statistical analyses were performed with SPSS statistical software (release 15.0). The composed weighting factor was used throughout all calculations. We present weighted results, unless stated otherwise. A probability level of p < 0.05 based on two-sided tests was considered statistically significant. No adjustments for multiple testing were made, due to the focus on survey effect.

## Results

### Characteristics of the study populations

Main characteristics of the study population are shown in Table 1. Survey populations were similar with respect to age, BMI, residence in West vs. East Germany, and selected pre-existing medical conditions, except for hyperlipidemia. Compared to women participating in the 1997–1999 survey, women surveyed in 2003–2004 were more likely to be current or former smokers and to be more physically active. Study populations also differed with respect to a higher proportion of women with lowest and highest social status in the more recent survey.

**Table 1:** Descriptive characteristics of women populations surveyed in German national health surveys before (1997–1999) and after (2003–2004) the publication of the EPT-WHI trial results

	**1997–1999 survey**	**2003–2004 survey**	**p-value**
**Sample size (unweighted total n)**	2284 (2248)	2362 (2215)	
***Continuous variables, mean ± SD***			
**Age, years**	59.8 ± 12.0	60.0 ± 12.5	0.437
**BMI, kg/m^2^**	27.8 ± 5.1	27.6 ± 5.2	0.566
***Nominal and ordinal variables, n (%)***			
**Residence in former West Germany**	1762(77.1)	1841(77.9)	0.515
**Social status**			
*Lower*	691(31.8)	848(36.9)	< 0.001
*Intermediate*	1115(51.4)	1003(43.7)	
*Upper*	364(16.8)	446(19.4)	
**Smoking status**			
*Smoker*	405(18.4)	485(20.5)	< 0.001
*Ex-smoker*	349(15.9)	538(22.8)	
*Non-smoker*	1447(65.7)	1339(56.7)	
**Physical activity**			
*0 hrs/wk*	1277(58.4)	1135(48.6)	< 0.001
*< 1 hrs/wk*	268(12.2)	232(9.9)	
*1–2 hrs/wk*	377(17.2)	503(21.5)	
*> 2 hrs/wk*	266(12.2)	466(19.9)	
**Pre-existing medical conditions **(yes vs. no)			
**Diabetes**	217(9.6)	228(9.7)	0.891
**Hypertension**	868(38.2)	950(40.4)	0.128
**Hyperlipidemia**	751(33.0)	881(37.5)	0.001
**CVD**	385(16.9)	355(15.3)	0.124
**Current MHT use^#^, n (%)**	386(16.9)	238(10.1)	< 0.001
≤ 1 year (new starters)	71(3.1)	16(0.7)	< 0.001
1–3 years	81(3.5)	31(1.3)	< 0.001
> 3 years (long-term users)	230(10.1)	189(8.0)	0.014

All results are weighted to the distribution of main demographic characteristics (age, sex, residence in West or East Germany and level of urbanity) in the German population according to official population statistics at the time of the survey as well as in 2003.

p-values are based on t-tests for continuous variables or χ^2^-tests for dichotomous variables.

EPT-WHI = combined estrogen plus progestin therapy arm of the Women's Health Initiative randomized controlled trial; BMI = Body mass index; CVD = cardiovascular disease; hr(s)/wk = hour(s)/week; MHT = menopausal hormone therapy.

^# ^Information for duration of MHT use was missing in 4 users in the 1997–1999 survey and 2 users in the 2003–2004 survey.

### Current MHT use and user profiles in the two national health surveys

**Figure 1:**
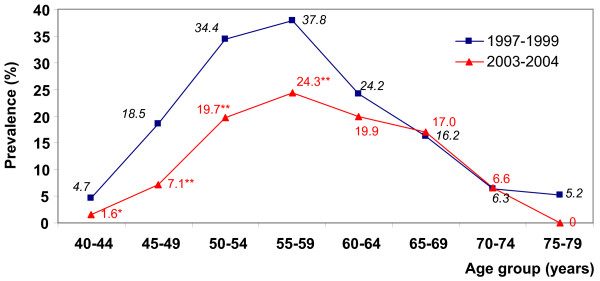
Age-classified current MHT use prevalence in the two German national health surveys conducted before (1997–1999) and after (2003–2004) the EPT-WHI study. MHT = Menopausal hormone therapy. EPT-WHI = combined estrogen plus progestin therapy arm of the Women's Health Initiative randomized controlled trial. * p < 0.05, ** p < 0.001, compared to age specific prevalence in the 1997–1999 survey.

A total of 419 (absolute number, unweighted) women were current MHT users in the 1997–1999 survey. Among 2003–2004 participants, 260 women were current and 392 were past MHT users (absolute number, unweighted). The overall weighted prevalence of current MHT use decreased by 40.2% from 16.9% of the sample in 1997–1999 to 10.1% in 2003–2004 (Table 1). In both national health surveys, the prevalence of current MHT users was curvilinearly related to age, with the highest rates among 50–59-year-old women (Figure 1 and Table 2). The difference in prevalence between surveys varied with age category. Absolute decreases as well as the percentage difference were largest among women 40–49 and 50–59 years of age as opposed to almost no difference among women 60–69 years of age (Table 2). A relative decrease of about 44% in current MHT use among women 70–79 years of age (Table 2) was attributable to differences among women 75 years of age and older (Table 2 and Figure 1). However, the number of current MHT users within this age group was very small (n = 18) in the 1997–1999 survey, and no woman in this 2003–2004 survey reported current use of MHT.

**Table 2:** Prevalence and determinants of current MHT use in the two surveys conducted before (1997–1999) and after (2003–2004) the publication of EPT-WHI trial results

	**Prevalence %**		**Difference %**		**OR (95% CI)**	
	
	**1997–1999**	**2003–2004**	**Absolute**	**Relative**	**1997–1999**	**2003–2004**
**Age, *years***						
*40–49*	10.7	3.9	-6.8	-63.6	0.20(0.15–0.27)	0.14(0.09–0.21)
*50–59*	36.3	21.3	-15.0	-41.3	1(Reference)	1(Reference)
*60–69*	20.3	18.5	-1.8	-8.9	0.56(0.42–0.76)	0.78(0.55–1.09)
*70–79*	5.7	3.2	-2.5	-43.9	0.13(0.07–0.23)	0.14(0.07–0.31)
**Region**						
*West Germany*	18.4	10.7	-7.7	-41.8	1.73(1.33–2.25)	1.74(1.15–2.64)
*East Germany*	11.9	7.9	-4.0	-33.6	1(Reference)	1(Reference)
**BMI**						
*< 25 kg/m*^2^	20.5	9.8	-10.7	-52.2	1.79(1.28–2.51)	1.38(0.90–2.11)
*25–30 kg/m*^2^	18.8	12.3	-6.5	-34.6	1.70(1.24–2.34)	1.42(0.96–2.10)
*> = 30 kg/m*^2^	10.0	7.5	-2.5	-25.0	1(Reference)	1(Reference)
**Social status**						
*Lower*	9.3	8.1	-1.2	-12.9	1(Reference)	1(Reference)
*Intermediate*	20.2	11.1	-9.1	-45.0	1.87(1.37–2.55)	0.85(0.61–1.18)
*Upper*	25.3	9.3	-16.0	-63.2	1.96(1.35–2.86)	0.73(0.48–1.10)
**Smoking status**						
*Smoker*	18.8	10.9	-7.9	-42.0	1(Reference)	1(Reference)
*Ex-smoker*	21.5	8.4	-13.1	-60.9	1.00(0.68–1.46)	0.91(0.60–1.38)
*Non-smoker*	16.1	10.5	-5.6	-34.8	0.86(0.64–1.17)	1.00(0.70–1.43)
**Physical activity**						
*0 hrs/wk*	13.5	8.5	-5.0	-37.0	1(Reference)	1(Reference)
*0–1 hrs/wk*	21.6	9.9	-11.7	-54.2	1.49(1.06–2.10)	1.15(0.71–1.88)
*1–2 hrs/wk*	22.0	11.1	-10.9	-49.5	1.29(0.94–1.76)	0.85(0.58–1.23)
*> 2 hrs/wk*	25.6	12.7	-12.9	-50.4	1.56(1.10–2.21)	1.02(0.71–1.48)
**Pre-existing medical conditions **(yes vs. no)						
**Diabetes**	6.4	3.5	-2.9	-45.3	0.74(0.41–1.33)	0.42(0.21–0.87)
**Hypertension**	14.3	9.9	-4.4	-30.8	1.06(0.80–1.39)	1.07(0.78–1.47)
**Hyperlipidemia**	17.3	11.4	-5.9	-34.1	1.03(0.79–1.33)	1.13(0.84–1.52)
**CVD**	9.4	10.1	+0.7	+7.4	0.80(0.51–1.25)	1.02(0.63–1.64)

Odds ratios (OR) and 95% confidence intervals (CI) were obtained from survey specific multivariable regression models with current MHT as the dependent variable. Explanatory variables included age (10-year age groups) as well as socio-demographic, behavioural and medical characteristics as shown above.

EPT-WHI = combined estrogen plus progestin therapy arm of the Women's Health Initiative randomized controlled trial; BMI = Body mass index; CVD = cardiovascular disease; hr(s)/wk = hour(s)/week.

The prevalence of 'new starters' declined significantly by 78% between survey periods (Table 1). A smaller percentage decrease (21%) was observed for the prevalence of long-term MHT users. However, the proportion of long-term users among women currently using MHT significantly increased by one third from 60.2% in 1997–1999 to 80.1% in 2003–2004 (p < 0.001, data not shown in Table 1). Among MHT users 60 years and older, the proportion of long-term users increased from 83.6% in 1997–99 to 96.4% in 2003–2004 (data not shown in Table 1).

Apart from age group, variables independently related to current MHT use in the 1997–1999 survey included residence in West Germany, higher social status, lower BMI and more health-conscious behaviour (Table 2). In the 2003–2004 survey, only associations with age category and residence in West vs. East Germany persisted. In particular, higher social status was significantly related to the prevalence of current MHT use in the earlier survey, but the association was no longer significant in the more recent survey (Table 2). This was also true for the prevalence of 'new starters' and long-term MHT users (data not shown). A significant and inverse association between current MHT use and a medical history of diabetes mellitus was observed in the 2003–2004 survey (Table 2).

MHT use was consistently higher in West than East Germany, but the absolute decrease was considerably larger among women living in the West. Similar patterns of highest MHT use in 1997–1999 and strongest decreases in use from 1997–1999 to 2003–2004 were observed in association with lower BMI, higher social status, higher level of physical activity, and ex-smoker status (Table 2).

### Impact of survey period on current MHT use among women in Germany

Pooling data from both surveys, we estimated the independent effect of survey period (2003–2004 vs. 1997–1999) on current MHT use in a MLR regression model adjusting for age, BMI, social-status index as well as health-related characteristics and pre-existing medical conditions. When first order interactions of co-variables with the survey variable were added to the full model, only the interaction with social-status index was highly significant (p = 0.001). This was also true when age was added as a dichotomous (< 60 vs. ≥ 60 years) co-variable (data not shown). Table 3 shows the results of the full model including the interaction between survey and social-status index. Altogether 20% of variability in current MHT use was explained by the model. As reflected by the estimates for the survey period and the interaction between survey period and social-status index, current MHT use was significantly less likely in 2003–2004 compared to 1997–1999 only among women of higher social status (Table 3). This finding was confirmed in MLR analyses stratifying by social status; the strength of the association between survey period and current MHT use showed a gradual decrease from highest (OR 0.24, 95% CI 0.15–0.38) via intermediate (0.44, 0.34–0.58) to lowest (1.01, 0.69–1.49) social-status classification.

**Table 3:** Odds ratios and 95% confidence intervals for current MHT use in the pooling regression model

	**β**	**p-value**	**Odds ratios and 95% confidence intervals**
**Age, *years***			
*40–49*	-1.767	< 0.001	0.17 (0.13–0.22)
*50–59*			1(Reference)
*60–69*	-0.392	< 0.001	0.68(0.54–0.85)
*70–79*	-2.099	< 0.001	0.12(0.09–0.17)
**Region**			
*West Germany*	0.540	< 0.001	1.72(1.34–2.20)
*East Germany*			1(Reference)
**Smoking status**			
*Smoker*			1(Reference)
*Ex smoker*	-0.071	0.643	0.93(0.69–1.26)
*Non-smoker*	-0.074	0.560	0.93(0.73–1.19)
**Physical activity**			
*0 hrs/wk*			1(Reference)
*0–1 hrs/wk*	0.218	0.154	1.24(0.92–1.68)
*1–2 hrs/wk*	0.151	0.241	1.16(0.90–1.50)
*> 2 hrs/wk*	0.275	0.039	1.32(1.01–1.71)
**Pre-existing medical conditions **(yes vs. no)			
**Diabetes**	-0.808	0.001	0.45(0.27–0.73)
**Hypertension**	0.099	0.364	1.10(0.89–1.37)
**Hyperlipidemia**	0.080	0.436	1.08(0.89–1.33)
**CVD**	0.012	0.937	1.01(0.75–1.38)
**BMI, ***kg/m*^2^	-0.052	< 0.001	0.95(0.93–0.97)
**Social-status index**	0.057	0.001	1.06(1.03–1.09)
**Survey period**^#^			
*1997–1999*			1(Reference)
*2003–2004*	0.556	0.076	1.74(0.94–3.22)
Social-status index * survey period	-0.107	< 0.001	0.90(0.86–0.94)

Odds ratios (OR) and 95% confidence intervals were obtained from multivariable regression models based on pooled data from both surveys with current MHT as the dependent variable and survey period as the main independent variable of interest. Co-variables included all other variables as shown above.

^#^: The OR for the survey period 2003–2004 vs. 1997–1999 is dependent on social-status index and is given by exp ^(0.556-social-status index*0.107) ^because of the significant interaction of social-status index with survey period. For example, given a low social-status index of 5, the OR for survey period 2003–2004 vs. 1997–1999 would be calculated to exp ^(0.556-5*0.107) ^= 1.02.

MHT = menopausal hormone therapy; CVD = cardiovascular disease; hr(s)/wk = hour(s)/week, BMI = Body mass index

## Discussion

### Main findings

In this analysis of two German national health surveys conducted before and after the publication of EPT-WHI results, we found that the overall prevalence of current MHT use among women aged 40–79 years decreased by 40.2% from 16.9% of the sample in the 1997–1999 survey to 10.1% in the 2003–2004 survey. Differences were most pronounced in subgroups of women in which MHT use was particularly common in the pre-WHI era. These included women 50–59 years of age, women residing in West Germany, and women with a lower BMI, more favourable health-related behaviour or higher social status. Apart from age category and residence in Western Germany, none of these characteristics continued to be associated with current MHT in 2003–2004, resulting in profound differences in user profiles. In particular, we observed a levelling of current MHT prevalence (overall current MHT users as well as 'new starters' and long-term users) between women of different social status. MLR analyses based on pooled data from both surveys confirmed that differences in current MHT use in 2003–2004 compared to 1997–1999 were modified by social status. A significant decrease in current MHT use was evident among women of higher but not of lower social status.

### Comparison with previous studies

The observation of a significant decline in MHT use among women in Germany is in accordance with results from previous studies conducted after the release of WHI results in the US [9-16] and various other European countries [17-24] including a regional study in Germany [26]. Our findings that the overall prevalence of current MHT among German women dropped to only 10% within 1–2 years after the publication of the EPT-WHI trial results is very much in accordance with recent reports from the United Kingdom and Italy [17,18]. A significant reduction in the prevalence of 'new starters' (women using MHT for up to one year) as observed here and elsewhere [35] lends further credence to the assumption, that the release and communication of study results had a major impact on MHT use among German women. The prevalence of long-term current users showed a smaller albeit still significant decline between surveys, while the relative proportion of long-term users increased. This probably reflects that long-term MHT users are less likely to quit MHT in the post-WHI era, and previous reports from other countries support this assumption [21,36]. In the present analysis, the proportion of long-term users among women on current MHT was significantly higher among women who were 60 years and older, compared to those of younger age groups. This may explain why we found significant decreases in current MHT only among women up to an age of 60 years. Results from previous studies regarding age specific patterns of decline in MHT are conflicting [21,37,38], but findings very similar to ours have been reported from the Netherlands [21,38].

In Germany, two studies have previously examined the association of WHI trial results with MHT use [25,26]. A randomly selected sample of 10,030 German women 45 to 60 years was contacted in a telephone survey in July 2003, and 59.9% completed an interview on prior MHT use. Overall, 88.6% of ever-users of MHT were aware of the WHI study results. Among these, 25.7% of women indicated having stopped MHT after the WHI study, whereas 14.2% had already discontinued MHT before the WHI study [25]. The prevalence of lifetime MHT use among women participating in this previous study was 35.8% [25], which is consistent with 33.9% of ever-users within the same age range in our 2003–2004 telephone survey (data not shown in the results). Estimates regarding the change in prevalence of MHT use could not be derived from the previous study, as information regarding duration and time of discontinuation of MHT was not systematically collected [25]. Within a regional health study of peri- and postmenopausal women 45–65 years of age, Clanget and colleagues observed a 16% decline in the prevalence of current MHT use (35.4% vs. 29.8%) between April 2000 and February 2003, while the prevalence of past MHT use increased accordingly (19.8% vs. 23.5%) [26]. Recruitment following the publication and media coverage of the WHI trial results extended only over a few months, which may explain why the observed differences were much less pronounced than in the present study.

Few studies have previously investigated the association of the WHI study results with MHT use according to educational level, professional status or household income. Most of these did not find any evidence for a modifying effect of socioeconomic background [16,17,39]. However, declines in MHT use in the post-WHI era have been observed to differ in US study populations of different educational background, with smaller changes reported in a less well-educated population of women enrolled in a Medicaid program [40] than in an unselected national sample of women [12]. A recent retrospective analysis of MHT use among women of a British birth cohort found the reverse; the prevalence of MHT use declined most among women with lower educational background [23]. Unlike the present study and a recent report from Italy [17], these authors also found a large decrease in MHT use among women with cardiovascular risk factors, in particular obesity and hypertension. These conditions are likely to correlate with lower social status; multivariable analyses were not performed, hence confounding could explain the apparent contradiction to our results [23].

We explored several possible explanations for the observed convergence of MHT use among women of higher social status with pre-existing patterns among lower social-status women. First, women from different social backgrounds may differ with respect to motivation for MHT use. As observed in our 1997–1999 survey, epidemiological studies have consistently found that MHT users characteristically differ from non-users with respect to a higher educational or socioeconomic status [17,41,42], lower BMI [41-44], and more health-conscious behaviour, specifically physical activity [41,42,44-46]. Kuh and colleagues previously suggested that educated women are more likely to start MHT for long-term prevention, as opposed to less-well educated women who use MHT as a remedy for symptoms and pre-existing health problems, such as an early menopause or prior hysterectomy [47]. Considering the fact that MHT was among the most heavily promoted 'anti aging' or 'health conserving' medications prior to the WHI study [11], it is conceivable that women of higher social status were using MHT mainly for the prevention of chronic conditions in later life, and would then be more likely to stop treatment following the release of the WHI results [9,36].

Unfortunately, we had no information on reproductive history and menopausal symptoms, which precluded a comparison of motivation and indications between MHT users of different social status in the two surveys. We can therefore not exclude that higher hysterectomy rates among women with lower social status confounded our observation of a larger decline in MHT use in association with higher social status. There is evidence for an inverse association between hysterectomy and social status from several epidemiological studies [48], including a survey conducted in 2000 among women in one metropolitan area of West Germany [49]. While these data are not representative at the national level, reanalysis of data from an earlier 1990–1991 national health survey in Germany showed that a self-reported history of hysterectomy used to be significantly more common among women of lowest social status compared to women with intermediate or highest social status (OR 2.00, 95% CI 1.50–2.67, Robert Koch-Institute, unpublished data). Hysterectomy rates were also significantly higher among West vs. East German women (25.4% vs. 18.6%, p = .002, for women 40–69 year-old, Robert Koch-Institute, unpublished data), which could explain why we observed a consistently higher prevalence of current MHT use in West vs. East Germany in the present analysis. It is possible that other health system specific differences between East and West Germany had a lasting influence on MHT use in the two parts of Germany. However, we cannot provide data to support any specific hypotheses. An association between hysterectomy and continuing MHT use has been reported by a number of studies, both in Europe and the US [9,12,24,50], although conflicting results also exist [25,51]. Consistent with this finding, lower discontinuation rates have been observed among women on estrogen monotherapy compared to those using estrogen/progestin combinations in the US [9,14-16,35,36] as well as in European countries [21,24,26].

Our findings concerning the connection between MHT use and social status may also reflect that social background influences women's information status on the risks and benefits of MHT. In their 2003 telephone survey, Heitmann and colleagues demonstrated a direct relationship between women's social status and their degree of information about the WHI study results [25]. In the North American Menopause Society 1998 menopause survey, women of lowest socioeconomic status were found to be least likely to have received counselling for postmenopausal MHT by medical professionals [52]. A recent nationally representative survey of 40–60-year-old US women found that less well-educated women were significantly less aware of WHI results and the impact of MHT on major chronic diseases [53]. It remains to be investigated whether women with lower social status are less well informed because they are less motivated to seek information or use different sources of information. It is also unknown whether health care provider factors (e. g. sex, age, communication skills or specifics of health care system organization, access to health care, source of information) are relevant. Health care organization as well as communication of scientific results within medical professional societies and coverage of health issues by the public media differ from country to country. Thus, it is possible that study results on the risks and benefits of MHT use had a different impact on specific subgroups of women in different countries. This may partly explain the conflicting results of previous reports on post-WHI MHT use and social status [16,17,39,40].

### Strengths and limitations of the present study

Both surveys were independent, highly standardized and population-based representative national health surveys, using largely identical core questionnaires and computer-assisted interview methods. In order to achieve comparability between surveys and to be able to extend the observations to the population level, survey results were weighted by a composite demographic weighting factor. This factor adjusted for deviations in main demographic characteristics between the study populations and official population statistics at the time of the specific surveys (1997–1999 and 2003–2004 respectively); in addition the factor integrated a comparison with 2003 population statistics as a common standard for both surveys. Societies change over time. Thus, survey research needs to be adjusted for inflation with regard to key socio-demographic characteristics. We therefore used a construct variable to describe social status, which was computed from identical component information regarding education, income and professional status, but was adapted to societal changes over time. Due to cohort effects, such adjustment will not remove all the differences between population samples of the same age range compared in repeat surveys over time. In our study, this was reflected by the fact that women of the two population samples still somewhat differed with respect to social status as well as health-related behaviour. We therefore adjusted for these variables in multivariable analyses. Previously conducted non-response analyses demonstrated that participation tended to be associated with a younger age and higher educational background in both surveys, whereas persons with pre-existing medical conditions were more likely to be overrepresented only in the 2003–2004 telephone survey [28,34]. However, the prevalence of pre-existing conditions with relevance to current MHT was similar among women of both surveys.

Our study has a number of limitations. First, our observations are only based on measurements at two points in time, and it would have been much preferable to support them by being able to assess intervening trends. However, the only other national health telephone survey conducted between the two surveys analyzed here, did not collect information on MHT use. Secondly, the 1997–1999 and 2003–2004 surveys differed with respect to sampling and interview methods, which could have biased our results. Most importantly, the validity of our outcome variable (current MHT use) may differ between surveys. While information on MHT in 1997–1999 was obtained in the context of a detailed personal interview on all current medications, during the 2003–2004 telephone survey interview women were specifically questioned about current as well as past MHT use. Thus it is possible, that MHT users in the first survey were more likely to be missed, the consequence of which would be an underestimation of initial MHT use and dilution of the difference between the two surveys. Misclassification with the same effect of decreasing the difference between survey periods could also result from over-reporting of current MHT use in the 2003–2004 survey, for example by women who had replaced MHT by complementary alternative medicine. As we found a strong overall decline in current MHT use between the two survey periods, our results would only be compatible with differential misclassification in association with lower social status. However, this seems unlikely, because significantly lower MHT use in association with lower social status as observed in the 1997–99 survey has been a consistent finding in previous studies of MHT [17,41,42], and there is no evidence for selective over-reporting of current MHT use among women with low social status.

## Conclusion

Between 1998 and 2004 current use of MHT considerably declined by 40% among women in Germany. While neither we nor others can prove a direct relationship to the interim publication and media coverage of results from the EPT-WHI trial and other studies such as the Million Women Study, this is suggested by parallel developments in various countries. Unlike previous reports, we observed profound differences in user profiles. In particular, decreases in the prevalence of current MHT use were most pronounced in women with higher social status. This resulted in a convergence of current MHT use among high social-status women with pre-existing patterns of use among women with low social status. Future studies should test the hypothesis that women of different social and cultural backgrounds differ with respect to motivation and indications of MHT use. International comparisons of MHT use and MHT-related health care patterns would help clarify the respective roles of patient, health care provider, and health system characteristics when translating the study results on the risks and benefits of MHT use into medical practice.

## Competing interests

The author(s) declare that they have no competing interests.

## Authors' contributions

YD performed the statistical analysis, conducted the literature review and drafted the original manuscript. MD provided specific knowledge and assisted in the conceptualization of the study design, the interpretation of results, and contributions to the final manuscript. HUM provided specific knowledge and assisted in the conceptualization of the study design and interpretation of study results. CSN assisted in the conceptualization of the study and provided assistance in analyzing the data, interpreting the results and writing the final manuscript. HK conceptualized and supervised the study. All authors read and approved the final manuscript.

## Pre-publication history

The pre-publication history for this paper can be accessed here:


